# Molecular dynamics and principal components of potassium binding with human telomeric intra-molecular G-quadruplex

**DOI:** 10.1007/s13238-015-0155-3

**Published:** 2015-04-18

**Authors:** Zhiguo Wang, Ruping Chen, Ling Hou, Jianfeng Li, Jun-Ping Liu

**Affiliations:** Institute of Aging Research, School of Medicine, Hangzhou Normal University, Hangzhou, 311121 China; School of Medicine, Shandong University, Jinan, 250100 China; Department of Immunology, Central Eastern Clinical School, Monash University, Melbourne, VIC 3004 Australia; Center for Cancer Research, Monash Institute of Medical Research, Clayton, VIC 3168 Australia

**Keywords:** G-quadruplex, potassium ion, molecular dynamics, principal component analysis, MM-PBSA

## Abstract

Telomere assumes intra-molecular G-quadruplex that is a significant drug target for inhibiting telomerase maintenance of telomeres in cancer. Metal cations have been recognized as playing important roles in stabilizing G-quadruplex, but their binding processes to human telomeric G-quadruplex remain uncharacterized. To investigate the detailed binding procedures, molecular dynamics simulations were conducted on the hybrid [3 + 1] form-one human telomeric intra-molecular G-quadruplex. We show here that the binding of a potassium ion to a G-tetrad core is mediated by two alternative pathways. Principal component analysis illustrated the dominant concerted motions of G-quadruplex occurred at the loop domains. MM-PBSA calculations revealed that binding was energetically favorable and driven by the electrostatic interactions. The lower binding site was found more constructive favorable for binding. Our data provide useful information on a potassium-mediated stable structure of human telomeric intra-molecular G-quadruplex, implicating in ion disorder associated conformational changes and targeted drug design.

**Electronic supplementary material:**

The online version of this article (doi:10.1007/s13238-015-0155-3) contains supplementary material, which is available to authorized users.

## INTRODUCTION

Telomere G-quadruplex consists of guanine-rich nucleic acid sequences characteristic of a four-stranded structure at chromosomal ends (telomeres) of eukaryotic cells (Moyzis et al., 1988). G-quadruplex exists in several π-π stacked G-tetrad layers, with each having four coplanar guanines that are held together through eight Hoogsteen hydrogen bonds (Burge et al., 2006; Williamson, 1994). Human telomeric DNA is double-stranded for most of its length with a terminal single-stranded overhang of 100–200 bases at the extreme 3′-end that has the propensity to fold into intra-molecular G-quadruplex structures under physiological ionic conditions (Burge et al., 2006; Makarov et al., 1997; Neidle and Parkinson, 2003). The intra-molecular G-quadruplexes have been recognized as significant drug targets by inhibiting the activity of telomerase that is overexpressed and required in ~85% of cancers (Maizels, 2006; Neidle and Parkinson, 2003; Oganesian and Bryan, 2007; Paeschke et al., 2005). So, inhibition of telomerase by targeting the stabilization of intra-molecular telomeric G-quadruplex has become an attractive strategy for the development of novel anti-cancer drugs.

Information at the atomic level of the human telomeric intra-molecular G-quadruplex structures is essential for structure-based rational drug design. By applying NMR and X-ray crystallography techniques, human G-quadruplexes at telomeres, gene promoter regions of proto-oncogenes (c-myc, c-kit, bcl-2) and untranslated regions of mRNAs have been investigated (Bejugam et al., 2007; Dai et al., 2006; Dexheimer et al., 2006; Phat et al., 2007; Rankin et al., 2005; Siddiqui-Jain et al., 2002). For the human telomeric G-quadruplex, the single stranded G-rich overhang confers the intra-molecular G-quadruplex structures with different conformational topologies, stabilization of which is important in telomere-elicited signaling in aging and cancer.

Monovalent cations, mainly alkali metal ions and ammonium ions, are essential for G-quadruplex, because the ions are particularly sensitive to the structural folding of G-quadruplex involving in the formation of loop types and overall conformations (Gaynutdinov et al., 2008; Phan et al., 2006, 2007a, b; Pinnavaia et al., 1978; Rujan et al., 2005). KCl and NaCl associate with parallel and anti-parallel G-quadruplexes respectively (Gaynutdinov et al., 2008; Phan et al. 2007a, b). Folding of the hybrid [3 + 1] form-one and form-two of human telomeric G-quadruplexes is facilitated by K^+^ (Bončina et al., 2012; Li et al., 2009), whereas unfolding is accompanied by potassium release from human telomeric DNA d[AG_3_(TTAGGG)_3_] (Bončina et al., 2012). A hierarchy potencies of the monovalent cations in stabilizing G-quadruplex is K^+^ > NH_4_^+^ > Na^+^, suggesting that a weak stabilizer can be substituted by a strong stabilizer in solution to stabilize telomeres.

Besides NMR and X-ray crystallography, computer based molecular dynamics (MD) has proven to be a powerful tool to investigate the characteristics of telomeric G-quadruplex at the atomic level (Akhshi et al., 2012; Špačková et al., 2001; Zhu et al., 2013). Two MD studies on the binding process of d[G_4_] and d[G_4_T_4_G_4_] G-quadruplexes to cations have been reported using the AMBER 4.1 software and PARM94 all atom force field (Fadrná et al., 2004; Špačková et al., 1999). However, the force field appeared inaccurate apparently in predicting the nucleic loop conformations. In addition, multiple binding pathways of cations to a two-layered 15-mer thrombin-binding quadruplex aptamer have been shown with the use of a sum of microsecond molecular simulation (Reshetnikov et al., 2011). Although monovalent cations especially K^+^ packed between G-tetrad layers have been accepted as a leading co-factor for the stability of G-quadruplex, little is known about the binding process of cations to any types of human telomeric intra-molecular G-quadruplex.

**Figure 1:**
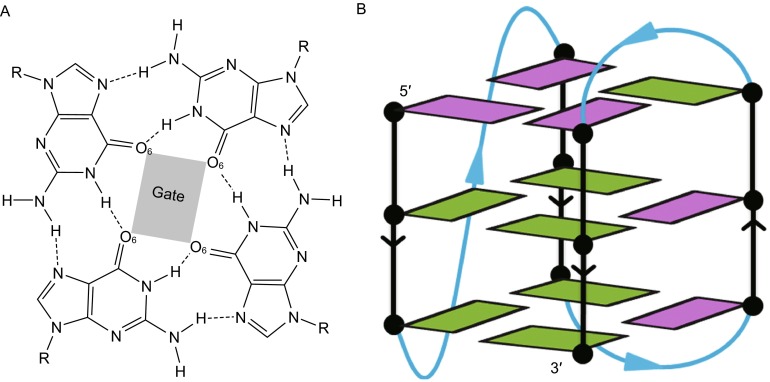
**Structure of human telomeric hybrid [3 + 1] form-one G-quadruplex**. (A) Top view of the top G-tetrad layer, Hoogsteen hydrogen bonds were denoted as dot lines; (B) side view of the whole schematic structure of G-quadruplex

To study the cation binding process to human telomeric d[TAG_3_(TTAGGG)_3_] that forms an intra-molecular antiparallel G-quadruplex, we carried out MD simulations on the interaction between potassium and G-quadruplex in solution. The G-quadruplex has three backbone strands in one direction and one strand in the opposite direction, and the hybrid [3 + 1] G-tetrads are in turn connected by the double-chain-reversal, edge-wise and edge-wise loops (Fig. 1). To discriminate from another hybrid G-quadruplex with altered loop types, the structure analyzed here was named as hybrid [3 + 1] form-one G-quadruplex. AMBER12 software and parmbsc0 combined FF99SB force field were applied, which has been demonstrated to provide closest G-quadruplex loops conformations and valued for DNA and G-quadruplex in many of nanoseconds state-of-the-art molecular dynamics simulations in aqueous solution (Fadrná et al., 2009; Pérez et al., 2007; Zhu et al., 2013). In the present work, using the popular molecular mechanic Poisson-Boltzmann surface area (MM-PBSA) and principal component analysis (PCA), we identified the preferred binding site and the most dominant motions during binding process, shading a fresh light on potential mechanisms of regulating the stabilities of telomeric intra-molecular G-quadruplex by physiological ions.

## RESULTS

Binding process of the strong stabilizer K^+^ with human telomeric hybrid [3 + 1] G-quadruplex was calculated in two independent 300 ns MD simulations (denoted as sim-K1 and sim-K2). The time scale is necessary for the monitoring of binding process and sampling conformations as previously suggested (Reshetnikov et al., 2011; Zhu et al., 2013).

Two pathways corresponding to the top and bottom G-tetrad gates in human hybrid [3 + 1] form-one G-quadruplex were recorded. The gates of central channel were constructed by electronegative O6 atoms of the top and bottom G-tetrad guanines (Fig. 1), and through these gated pathways cations migrated into the central channel of G-quadruplex. The top gate was capped by T1, A2 and the second edge-wise loop bases, and the bottom gate was half capped by bases of T12, T13 and A14. The loop caps prevented direct interactions between G-tetrads and K^+^ ions from environment, suggesting that the loop bases blocking the pathways should realign to permit an access of cations to the central channel.

### Potassium capture in sim-K1

**Figure 2:**
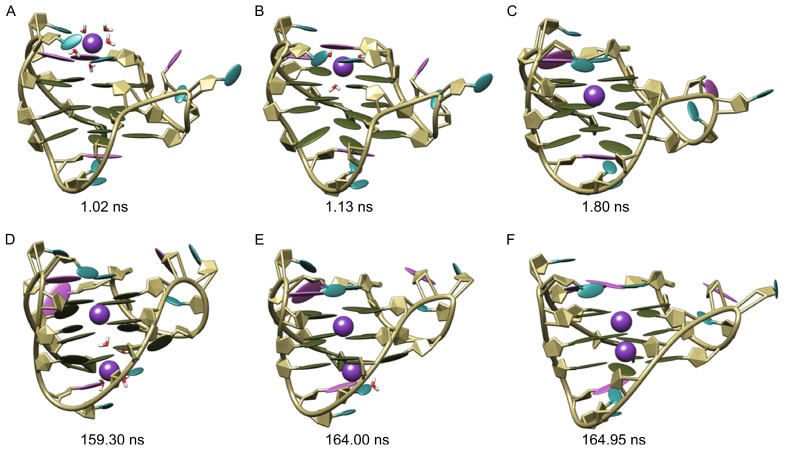
**Binding process of potassium ions with human telomeric hybrid [3 + 1] form-one G-quadruplex in sim-K1**. The bases of adenine, thymine and guanine nucleotides were colored orchid, cyan and olive green, respectively; potassium ions were represented as sphere and colored purple

Two K^+^ ions were located in the channel of G-quadruplex. The first K^+^ ion became initially bound at about 1 ns and reached quickly to the upper binding site between the top and central G-tetrads. While the second K^+^ ion reached the lower binding site between the bottom and central G-tetrads through the bottom pathway, it bound at 159.3 ns and spent about 5.6 ns to accomplish the binding process. The key structures during the binding process were extracted and presented in Fig. 2. The first K^+^ bound with the top cap nucleotides at 1.02 ns, and the base of T18 nucleotide in the second edge-wise loop tilted outward to accommodate the hydrated K^+^ ion (Fig. 2A). K^+^ became dehydrated and passed through the hindrance of cap residues to the space between the cap and top G-tetrad (Fig. 2B). In this process, there was a transient twist of the C3′-O3′ bond in the nucleotide T1 with the ε torsion angle changed from ~170° to 69°, and the bonds of the C1′-N1 between T1 and C1′-N9 in A2 were also twisted to create more space for the passage. The K^+^ quickly became completely dehydrated and arrived at the upper binding site at 1.80 ns (Fig. 2C).

With one K^+^ bound the G-quadruplex was stable, without apparent conformational changes detected till the second K^+^ beginning binding to the bottom cap at 159.30 ns (Fig. 2D). The second K^+^ approached to the binding site by the *re* face of the bottom cap nucleotides, where G5 was partly exposed to water bulk. In this process, two water molecules became dehydrated, the C3′-O3′ bond in T13 was twisted with the ε torsion angle changed from ~119° to 90.7°, and the base parts of G5 and T12 were temporarily rotated by the electrostatic interactions between K^+^, O6 in G5 and O4 in T12. Bound K^+^ became further dehydrated to get closer to the center of bottom G-tetrad gate (Fig. 2E), and finally arrived at the lower binding site at 164.95 ns by losing all of its coordinated waters. The resulting G-quadruplex with two K^+^ ions bound seems to be quite stable benefited from internal electrostatic interactions.

### Potassium capture in sim-K2

**Figure 3:**
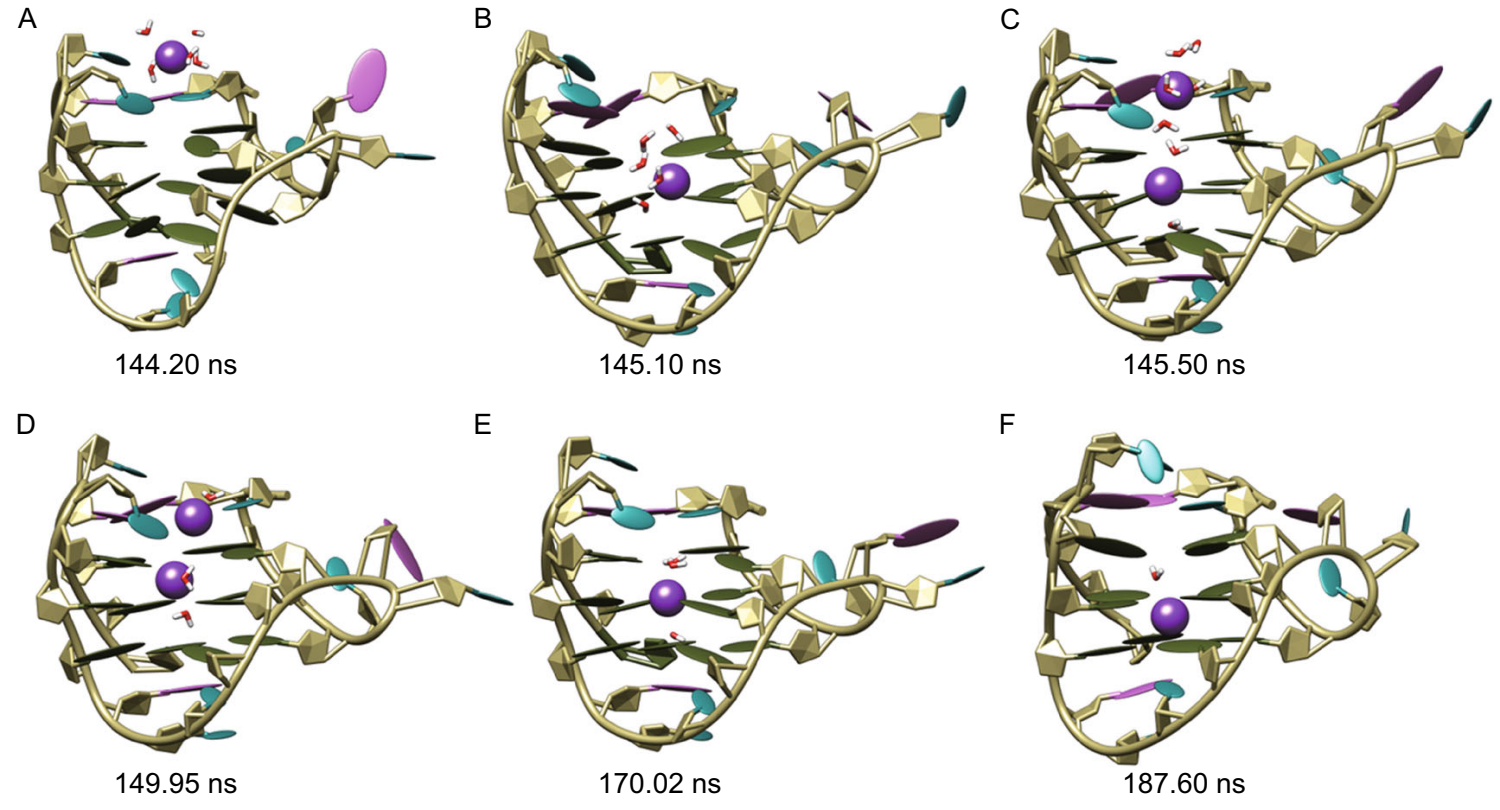
**Binding process of potassium ions with human telomeric hybrid [3 + 1] form-one G-quadruplex in sim-K2**. The bases of adenine, thymine and guanine nucleotides were colored orchid, cyan and olive green, respectively; potassium ions were represented as sphere and colored purple

The binding process of K^+^ with G-quadruplex in the sim-K2 was shown in Fig. 3. Probably due to the difference of the starting structures (Reshetnikov et al., 2011), the initial binding that occurred at 144.20 ns around the top pathway was significantly later than that in sim-K1 (Fig. 3A). The conformational changes caused by initial binding were similar to that caused by the binding of first K^+^ ion in sim-K1, except that the twisting of C3′-O3′ bond in nucleotide T1 was larger (the ε torsion angle changed from ~170° to 20.3°) resulting in a bigger channel entrance to allow relatively less dehydrated K^+^ ion to pass through the cap hindrance (Fig. 3B). This suggests the probable mechanism by which the bulky K^+^ with five water molecules bound at the upper binding site at 145.10 ns. In addition, the cap entrance was kept open by the coordinated water molecules thereby generating a broad passage directly linking the top G-tetrad gate to environment.

The open state of the cap entrance lasted for about 4.8 ns, during which a second K^+^ ion was found to be captured through the same pathway as the first one (Fig. 3C and 3D). However, it was unable to pass through the top gate that was held firmly by the first K^+^ ion, and was finally compelled back to environment (Fig. 3E). Then the top pathway entrance was closed, the remaining K^+^ with three coordinated waters oscillated between the upper binding site and the geometric center of central G-tetrad layer during the period from ~146 ns to ~187 ns. With time, two waters (the lower water and one of upper coordinated waters) escaped to the bulk from the bottom G-tetrad gate and the wide groove respectively. Finally, by passing through the central G-tetrad, the K^+^ reached the lower binding site at 187.60 ns, a conformation achieved till the end of observation (Fig. 3F).

### RMSD and G-tetrad gate area

**Figure 4:**
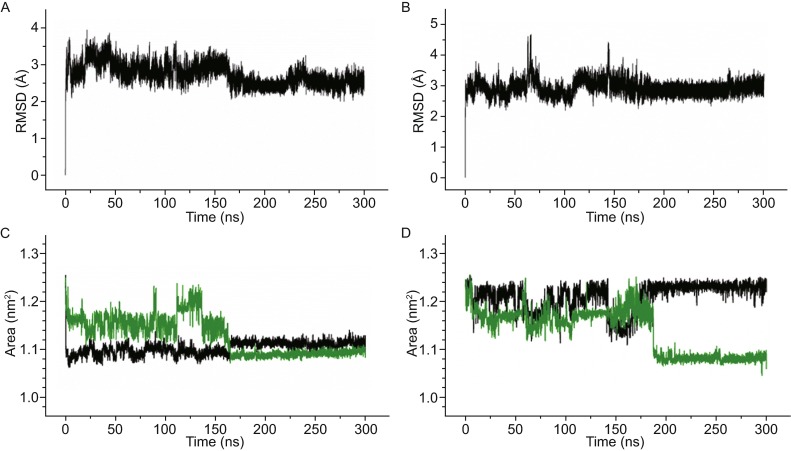
**The RMSDs and the fluctuations of G-tetrad gate areas**. (A and B) represent the RMSDs of sim-K1 and sim-K2, respectively; (C and D) represent the fluctuations of G-tetrad gate areas, in which the top and bottom gate area were shown as black and green lines, respectively

Root mean square deviation (RMSD) of the G-quadruplex reveals the entire structural fluctuations of a nucleotide structure throughout the MD simulation. The RMSDs of two simulations were given in Fig. 4A and 4B. We observed no drastic fluctuations with long smooth flat RMSD curves over 125 ns, suggesting that the resulting G-quadruplexes are considerably stable with no significant changes. Fluctuations seem originated from the effects of water solvent and environmental K^+^ ions and the bound K^+^ ions that affect involving atoms during the binding process. To understand the directly involved residues in the binding process, the areas of top and bottom G-tetrad gates constructed by their respective guanine O6 atoms were monitored (Fig. 1), using the molsurf module in AMBER12. The presence or absence of K^+^ inside the G-tetrad core affected the gate areas through electrostatic interactions (Fig. 4C and 4D).

In sim-K1, the area of top G-tetrad gate showed a sudden decrease at the starting time, since G-quadruplex get initial binding by the first K^+^ ion at 1.02 ns. At the meantime, the bottom gate area also decreased slightly by the relatively weak electrostatic attraction (Fig. 4C). The area of top gate did not vary significantly till the second K^+^ ion moved to the inside of bottom cap at 164 ns. While the top area became increased by about 0.04 nm^2^ due to the upward shift of the first K^+^ ion pushed by electrostatic repulsion from the second K^+^ ion, the bottom G-tetrad gate area decreased significantly by the binding of the second K^+^ ion through bottom pathway. The temporary increase in the bottom G-tetrad gate area between 111.64 ns and 136.59 ns corresponded to the inward tilt of G5 and G11 bases, the Hoogsteen hydrogen bonds between G5 and G11 bases destroyed in this period, so the structures were instable and reverted to the common form. No matter the G-quadruplex is in a free form or becomes completely bound by two K^+^ ions, the top gate area appeared larger than the bottom one, suggesting that the top gate is a preferred K^+^ binding pathway.

The large variations of gate areas before 144.20 ns in sim-K2 corresponded to transient binding of K^+^ ions to the outside of G-quadruplex gates and subsequent quick dissociation. Accompanied with the effective binding of K^+^ through the top pathway, the top gate area became drastically decreased at about 145 ns and achieved to a minimum at about 150 ns as the coordinated water dehydrated (Fig. 3D and 4D). Then the top gate area gradually increased and finally recovered close to the initial size in a synchronized manner with the location shift of bound K^+^ ion from the upper binding site to the lower binding site (Fig. 3E and 3F). In contrast, the lower gate area increased by the tilt of G5 and G11 bases as the K^+^ ion shifted, and a sudden decrease occurred when the K^+^ achieved the lower binding site. The final area of the lower gate is 1.08 nm^2^, near identical to that of sim-K1.

### Role of coordinated water

**Figure 5:**
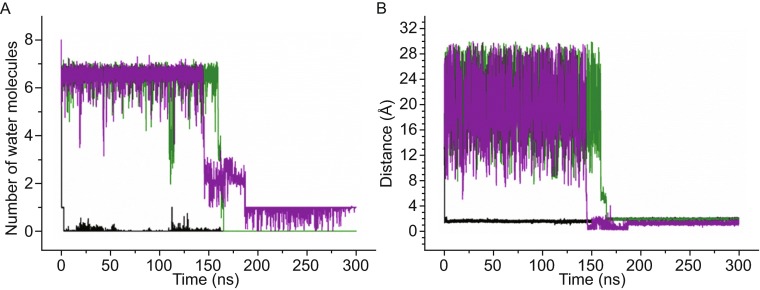
**The variations of coordinated waters and distances in sim-K1 and sim-K2**. For clarity, curves were smoothed by the adjacent-averaging method with a window of 10 points. (A) The number of waters within 3.5 Å of binding potassium ions; (B) the distances between the bound potassium ions and CCG. The variations in sim-K1 corresponding to the top and bottom pathway binding processes were respectively colored black and green, the variations in sim-K2 corresponding to the top pathway binding processes were colored purple

Although water molecules in the cation binding process to nucleic acids play indispensable role, the roles of water molecules in K^+^ binding to human telomeres have not been elucidated. We found that the coordinated water molecules of the bound K^+^ ions participated in the whole binding processes in both MD simulations (Figs. 2 and 3). To unveil the effects of coordinated waters (within 3.5 Å of K^+^ ion) (Reshetnikov et al., 2011), fluctuations of coordinated water numbers of bound K^+^ were monitored as K^+^ binding proceeded, and their impacts on conformation were analyzed (Fig. 5A). At the meantime, the geometric center of the four O6 atoms in the central G-tetrad (CCG) was set as a reference, and the distances between K^+^ ions and CCG across the entire simulations (as a direct location indicator of binding K^+^ ions) were presented in Fig. 5B.

In sim-K1, the coordinated waters of the first bound K^+^ ion decreased significantly as the ion moved towards the CCG through the top pathway, with the number changed quickly from six to one at the beginning of MD simulation (Fig. 5A and 5B). This appeared in accordance with the binding process (Fig. 2), suggesting that cation binding and dehydration processes are synchronous. The last coordinated water molecule returned back to environment through the upside of the wide groove. As shown in Fig. 5A, the intermittent fluctuations of the curve (black) before 159 ns corresponded to one water molecule that occasionally accessed into the detection range. This water molecule formed weak hydrogen bonds with the top G-tetrad and was finally compelled back to environment leading to a more compact G-quadruplex structure. The binding of the second K^+^ ion also started with water dehydration at about 159 ns (Fig. 2D). Within a period of about 6 ns, the number of coordinated water molecules quickly changed to four, then to one and zero. The starting time and duration of the dehydration process agreed with the binding process of K^+^ ion. In sim-k2, the dehydration process was similar to that in simulation one, except for a much longer dehydration period (over 42 ns), and there was one coordinated water molecule retained in association with bound K^+^ till the end of MD.

The variations in distance between bound K^+^ ions and CCG were consistent with the binding and dehydration processes as shown in Fig. 5B. In sim-K1, the original distance between the upper binding site and CCG was ~1.58 Å, but when the second K^+^ ion achieved the lower binding site, the distance became ~1.96 Å. The increased distance was near identical to the distance between the lower binding site and CCG, suggesting a symmetrical-like binding conformation along the central G-tetrad plane. In sim-K2, while there was only one bound K^+^ ion, the traversing conformations corresponded to the zero values in distance (purple curve) (Fig. 3E). The subsequent distance between the binding site and CCG was ~1.39 Å.

### Conformational alternations

**Figure 6:**
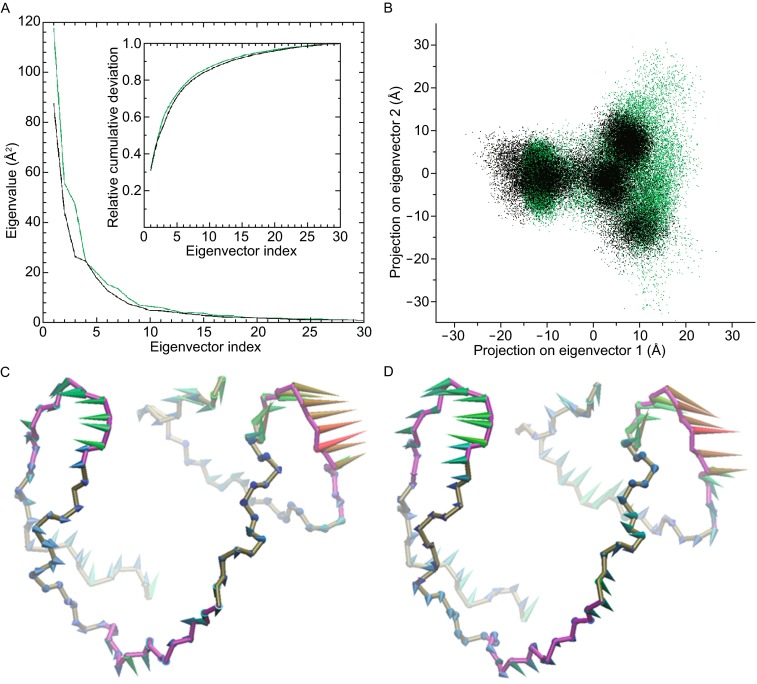
**Principal component analysis**. (A) Eigenvalue profiles constructed by the first 30 eigenvectors from PCA analysis of sim-K1 and sim-K2, which were represented by black and green lines, respectively. (B) two-dimensional projection of backbone atomic trajectories along the first two eigenvectors, projections in sim-K1 and sim-K2 were represented by black and green dots, respectively. (C and D) respectively show the dominant motions along the first eigenvector in sim-K1 and sim-K2 by porcupine plot

Dominant concerted motions of G-quadruplex in both ensembles were investigated by PCA. The concerted motions among atoms are expressed by the eigenvectors of the covariance matrix, and the extent of concerted fluctuational motion is expressed by the corresponding eigenvalue. Previous studies have shown that overall fluctuations of macromolecule are accountable by a few low frequency eigenvectors with large eigenvalues (Zhu et al., 2013). The eigenvalues of the first 30 eigenvectors and their relative cumulative deviations of these two G-quadruplex ensembles were shown in Fig. 6A. The majority of conformational fluctuations (>75%) was due to the concerted motions specified by the first six eigenvectors. Contributions to the motions were specified by the first eigenvectors in sim-K1 and sim-K2 (31% and 32%, respectively) (Fig. 6A).

To further investigate the essential motions, we investigated two-dimensional projections defined by the first two eigenvectors of sim-K1 and sim-K2. Both projected trajectories were confined within certain regions (Fig. 6B), suggesting concerted motions. The main regions of the two projections superimposed well, indicative of G-quadruplex structures in similar dominant conformational alternations in the two independent MD ensembles (Fig. 6B). The directions and extents of conformational movements along the first eigenvectors of sim-K1 and sim-K2 were represented by arrows (Fig. 6C and 6D). The most flexible regions consisted of three connecting loops (colored purple), especially the double-chain-reversal and the second edge-wise loops (Fig. 6C and 6D). In both ensembles, the double-chain-reversal and the second edge-wise loops moved outwards, whereas the first edge-wise loop moved downwards (Fig. 6C and 6D). The similarity of the motion patterns suggests that G-quadruplexes in the two ensembles undergo comparable conformational changes, with the motions contributing to a compact structural conformation.

### Binding free energies

The binding free energies of G-quadruplex and K^+^ ions in sim-K1 and sim-K2 were summarized in Table 1. MM-PBSA method has been shown to be reliable in correlation with experimental results (Yang et al., 2011). Whereas the major driving force of binding energy is contributed by electrostatic interaction, polar solvation and van del Waals interactions build negative contributions. Consistently, our data showed that in sim-K1, the independent binding free energies from K^+^ ions to the upper and lower binding sites were −73.79 and −76.58 kcal·mol^−1^, respectively (Table 1). In sim-K2, the binding free energy was −35.01 kcal·mol^−1^, where the negative values indicated that the binding processes are energetically favorable. The difference in large binding energy between sim-K1 and sim-K2 may be caused by their conformational distinctions as the G-quadruplex structure in sim-K1 was more compact than that in sim-K2. Another significant factor for the energy difference was that the bound K^+^ ion in sim-K2 was incompletely dehydrated, suggesting unfavorable electrostatic interactions between K^+^ and G-tetrad O6 atoms. The energy difference of 2.79 kcal·mol^−1^ between lower and upper binding sites in sim-K1 indicated that the lower binding site was more energetically favorable for K^+^ binding, which may be the reason that the K^+^ in sim-K2 finally located at the lower binding site.

**Table 1:** Binding free energy calculations for the G-quadruplex-K^+^ ion systems

Calculations	ΔE_ele_	ΔE_vdW_	ΔG_PB,sol_	ΔG_np,sol_	ΔE_MM_	ΔG_sol_	TΔS	ΔG_bind_
sim1-K_upper_	−743.60	14.98	643.81	−0.61	−728.62	643.20	−11.63	−73.79
sim1-K_lower_	−748.20	14.15	647.31	−0.61	−734.05	646.70	−10.77	−76.58
sim2-K	−699.42	9.83	645.27	−0.61	−689.59	644.67	−9.92	−35.01

## DISCUSSION

The present study shows the detailed dynamic binding processes of potassium ions and human telomeric G-quadruplex. Our results showed that G-quadruplex recruits one or two K^+^ ions from environment and that, K^+^ destines at the central channel of the G-tetrads. The starting time of binding process varied significantly, which could be attributed to the different initial structures. The initial structures consist of G-quadruplex, K^+^ ions and environmental waters in both MD ensembles. Though the hybrid [3 + 1] form-one G-quadruplex structures share the same conformation, the potassium counterions added according to a rough Coulombic potential around G-quadruplex had different locations in sim-K1 and sim-K2, which leaded to the time difference of approaching to the binding sites. But once the K^+^ reaches the space between the caps and G-tetrad gates, binding occurs and finishes quickly. So the rate-limiting step would be effective initial binding of potassium. This conclusion is in consistent with the recent findings of cation binding to 15-TBA DNA duplexes (Reshetnikov et al., 2011).

Coordinated water molecules have been found to play an important role in the whole process of K^+^ binding to G-tetrads. The findings that partial dehydration occurred as the initial binding took place suggest that the spaces for binding between the caps and G-tetrads cannot accommodate fully hydrated K^+^ ion and dehydration is a prerequisite for K^+^ binding. In addition, we found that dehydration consistently started from the lateral coordinated waters of K^+^ by checking the interval conformations, and the traversing process started with the bottom coordinated water of K^+^ through the top pathway, or the top water through bottom pathway. So, the coordinated water molecules appear to play as precursors of K^+^ binding in the interior traversing process.

In validating our results with two additional MD simulations using Na^+^ as counterion named sim-Na1 and sim-Na2, the binding processes of G-quadruplex and Na^+^ shown in Fig. S1 confirmed our findings in that the binding processes of Na^+^ and K^+^ are similar, and binding of Na^+^ and K^+^ produces comparable effects on G-quadruplex conformations (Figs. S2 and S3). However, we found that the higher hydration ability of Na^+^ than that of K^+^ makes Na^+^ spend more time to accomplish the traverse process in both pathways from the top or bottom entry gates.

Recently Rajendran et al. succeeded in the detection and single-molecule visualization of the G-hairpin and G-triplex intermediates and have found that ionic concentrations are pivotal to the yield of G-quadruplex and intermediates (Rajendran et al., 2014). They showed that in the concurrent presence of 10 mmol/L Mg^2+^ and 100 mmol/L K^+^, the maximum yields of G-triplex (57%) and G-quadruplex (76%) were obtained. In our simulations, the concentration of K^+^ (~174 mmol/L), as calculated from the volume of octahedron box and the number of K^+^ ions, should allow the full occupation of the G-tetrad core by two K^+^ ions according to Rajendran’s analysis. Though our MD simulations may cover most key conformational changes of G-quadruplex upon K^+^ binding, the time scale limited by current computer hardware and affordable computing time cannot ensure that all simulations have reached their final state. So, our current MD simulations focused on the description of the binding process at atomic level. To recognize the overall functions of the K^+^ and Mg^2+^ in the folding mechanism of G-quadruplex, a number of MD simulations form different starting conformations and further investigations on the interactions of metal cations and G-quadruplex intermediates are still needed.

Our MD simulations and MM-PBSA calculations suggest that the two K^+^ binding sites are not equivalent and the lower binding site is energetically favorable. The major driving force in K^+^ binding to G-quadruplex is electrostatic interactions of K^+^ with G-tetrads. When designing G-quadruplex targeted drugs, molecules with respective specificities towards the top and bottom gates of K^+^ entries in human telomeric G-quadruplex might be a desirable strategy for improved bioactivities. In addition, the findings in this work may provide some new insight into the structural characteristics of human telomeric intra-molecular G-quadruplex under conditions of ion disorder or electrolyte disturbances s of severe illness of metabolic disorders.

## MATERIALS AND METHODS

### Structure preparation

The initial structure of human telomeric hybrid [3 + 1] form-one G-quadruplex was retrieved from PDB data bank with the entry ID of 2JSM (Phan et al. 2007a, b). There are ten sets of coordinates for each atom of d[TAG_3_(TTAGGG)_3_], only the first set was retained since they were well superimposed with each other. No cations were existed in this NMR detected structure. In order to investigate the binding process of K^+^ ions and keep system in electric neutrality, 22 potassium counterions were added around G-quadruplex by using the AMBER xleap module. The complex was further immersed into the center of a truncated octahedron box of TIP3P water molecules with a margin distance of 12.0 Å. According to this procedure, two independent models were created and subjected to molecular dynamics calculations. Moreover, models using Na^+^ as counterion were also built in the same way.

### Molecular dynamics simulations

In order to remove bad contacts between nucleotides and surrounding cations and water molecules, each model was energy minimized by steepest descent method for 2000 steps with AMBER sander module, during which K^+^ ions and water molecules were set free while all nucleotides of G-quadruplex were restricted by a harmonic constraint of 100 kcal·mol^−1^·Å^−2^. The system energy was further minimized by conjugate gradient method for 4000 steps with no constraint. Then the system was gradually heated in the NVT ensemble from 0 K to 300 K over a period of 200 ps using Langevin thermostat with coupling coefficient of 1.0 ps and a weak constraint of 10 kcal·mol^−1^·Å^−2^ on nucleotides, and the system was subjected to an equilibrium simulation for 200 ps by removing all constraints. Finally, a 300 ns production simulation for each model was conducted under NPT ensemble, during which the temperature was kept 300 K by using Berendsen thermostat method with time constant of 1 ps, isotropic constant pressure boundary condition was applied by using Berendsen pressure coupling algorithm with a time constant of 1 ps. Periodic boundary conditions were applied to avoid surface effects. Covalent bonds containing hydrogen atoms were constrained using the SHAKE algorithm, and the long-range electrostatic interactions were treated by the Particle Mesh Ewald (PME) method. Both of the cutoff distances for long-range electrostatic and van der Waals interactions were set to 10.0 Å, time step was set to 2 fs in all of the simulations, coordinates were saved every 1 ps to record the molecular dynamics trajectory.

### Principal component analysis

To identify essential degrees of freedom in molecular motions from molecular dynamics (MD) trajectories, principal component analysis was performed for production trajectory by removing the overall translational and rotational motions. Because PCA filters the main modes of collective motion from much more local fluctuations, which in many cases reflect the functions of proteins or nucleotides, PCA was also termed “essential dynamics”. The principle of PCA has been described in detail elsewhere (Amadei et al., 1993; Zhu et al., 2013), and it has been widely used in the MD studies of protein and nucleotides.

PCA analysis was carried out by using the method of Interactive Essential Dynamics (IED) with the ptraj module in AMBER12 (Mongan, 2004). The G-quadruplex backbone atoms of the whole 300 ns trajectory were selected for calculation and analysis. Trajectory projections on certain eigenvectors were conducted to show the time-dependent motions that the atoms perform in the corresponding vibrational modes, they can also help determine coupled motions between different eigenvectors. Porcupine plot was generated by using VMD software to show a graphical summary of the motions along a particular eigenvector (Humphrey et al., 1996).

### Binding free energy calculations

The binding free energies of potassium ions and G-quadruplex were obtained by using MM-PBSA method in AMBER12, snapshots of the last 100 ns with an interval of 200 ps were extracted for the calculation. The binding free energy (ΔG_bind_) can be computed by the free energy difference between binding complex (G_complex_) and the sum of G-quadruplex nucleotides (G_quad_) and ions (G_ion_) as follows:

1 $$\Updelta {\text{G}}_{\text{bind}} =_{{}} {\text{G}}_{\text{complex}} - \left( {{\text{G}}_{\text{quad}} + {\text{ G}}_{\text{ion}} } \right)$$

Each free energy term can be calculated according to the equation:

2 $${\text{G }} = {\text{ E}}_{\text{MM}} + {\text{ G}}_{\text{solv}} - {\text{TS}}$$

where E_MM_ is the molecular mechanics energy in gas phase, G_solv_ is the solvation free energy, and TS represents the entropy contribution. The E_MM_ term consists of internal strain energy (E_int_), van der Waals energy (E_vdW_) and electrostatic energy (E_ele_):

3 $${\text{E}}_{\text{MM}} = {\text{ E}}_{\text{int}} + {\text{ E}}_{\text{vdW}} + {\text{ E}}_{\text{ele}}$$

The solvation free energy is divided into a polar part (G_PB_) and a nonpolar part (G_np_):

4 $${\text{G}}_{\text{solv}} = {\text{ G}}_{{{\text{PB,sol}}}} + {\text{G}}_{{{\text{np,sol}}}}$$

Therefore, according to above equations, ΔG_bind_ can be expressed as:

5 $$\Updelta {\text{G}}_{\text{bind}} = \, \Updelta {\text{E}}_{\text{int}} + \, \Updelta {\text{E}}_{\text{vdW}} + \, \Updelta {\text{E}}_{\text{ele}} + \, \Updelta {\text{G}}_{{{\text{PB,sol}}}} + \, \Updelta {\text{G}}_{{{\text{np,sol}}}} - {\text{T}}\Updelta {\text{S}}$$

In equation 5, internal strain energy difference (ΔE_int_) equals zero for our system since no contribution difference of bond, angle and torsion takes place upon ion bonding. The polar part of solvation ΔG_PB, sol_ is calculated by solving the Poisson-Boltzmann (PB) equations with the value of 1 and 80 for the interior and exterior dielectric constants respectively (Gilson et al., 1988). The nonpolar part of solvation ΔG_np, sol_ is determined by the solvent accessible surface area (SASA) using AMBER molsurf module according to the following equation

6 $$\Updelta {\text{G}}_{{{\text{np,sol}}}} = \, \gamma \Updelta {\text{SASA }} + \, \beta$$

where surface tension γ and offset β were set to the standard 0.00542 and 0.92 kcal·mol^−1^, respectively. The entropy term TΔS was calculated with normal mode analysis using the NMODE module in AMBER12 (Case et al., 2005).

## Electronic supplementary material

Supplementary material 1 (PDF 294 kb)

## ABBREVIATIONS

MD: molecular dynamics
MM-PBSA: molecular mechanic Poisson-Boltzmann surface area
PCA: principal component analysis
sim-K1: the first molecular dynamics simulation of G-quadruplex with potassium ions
sim-K2: the second molecular dynamics simulation of G-quadruplex with potassium ions
sim-Na1: the first molecular dynamics simulation of G-quadruplex with sodium ions
sim-Na2: the second molecular dynamics simulation of G-quadruplex with sodium ions
