# Supplementary material for: Molecular dynamics and principal components of potassium binding with human telomeric intra-molecular G-quadruplex
Source: Protein Cell. 2015 Apr 18;6(6):423–33. doi: 10.1007/s13238-015-0155-3 (PMC4444812; doi:10.1007/s13238-015-0155-3)
Supplement: Supplementary file 1 — Supplementary material 1 (PDF 294 kb) [file 13238_2015_155_MOESM1_ESM.pdf]

Molecular Dynamics and Principal Components of Potassium Binding with Human Telomeric Intra-molecular G-Quadruplex

Zhiguo Wang [*,1], Ruping Chen [1,2], Ling Hou [1], Jianfeng Li [1], Jun-Ping Liu [*,1,2,3,4]

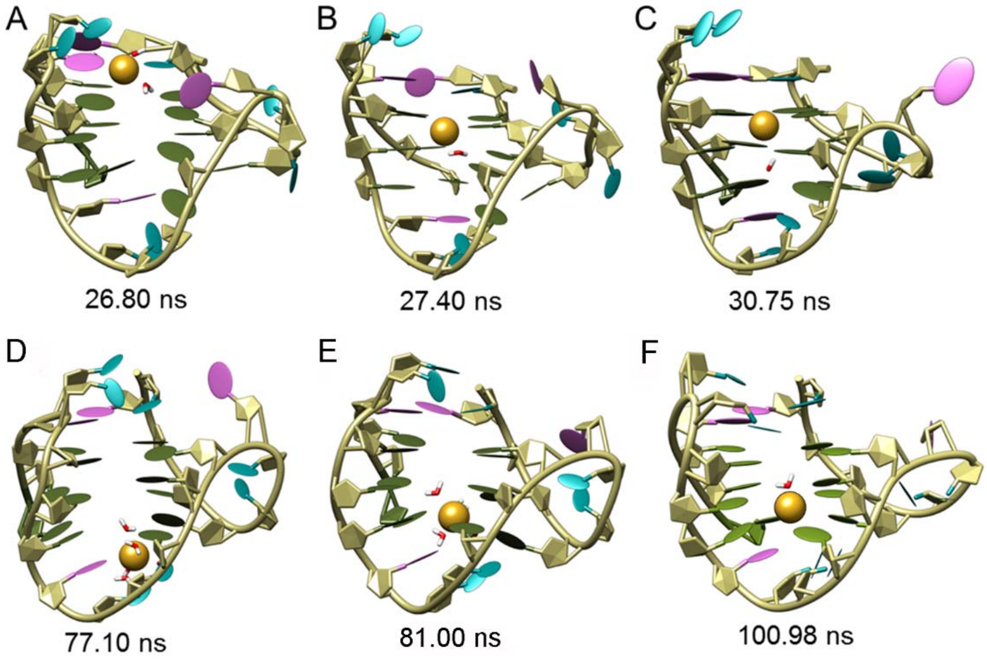

Figure S1

Binding processes of Na^+ ions with human telomeric hybrid [3+1] form-one G-quadruplex. Figures (A)-(C) represent the binding process through the top pathway in sim-Na1; (D)-(F) represent the binding process through the bottom pathway in sim-Na2. The bases of adenine, thymine and guanine nucleotides were colored orchid, cyan and olive green, respectively; the Na^+ ions were represented as yellow spheres.

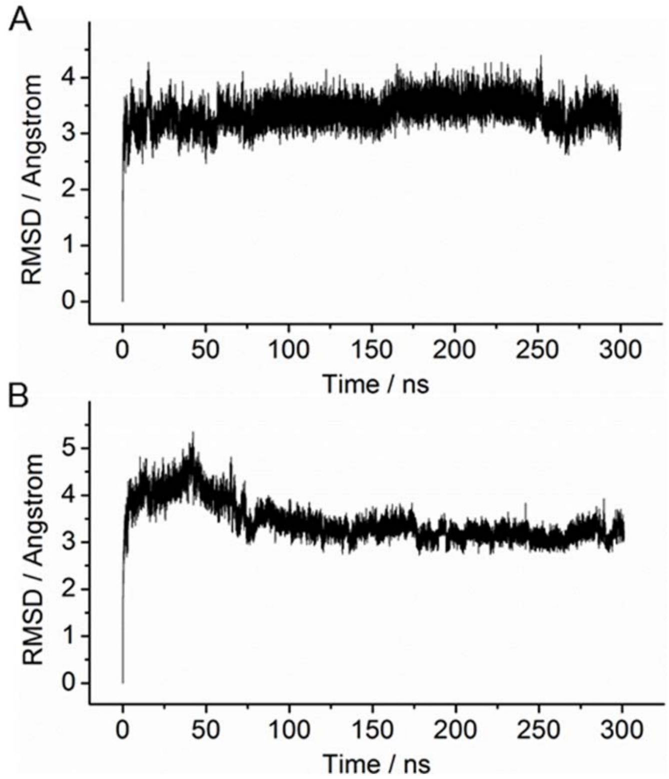

Figure S2

The RMSDs of human hybrid [3+1] form-one G-quadruplex in Na^+ containing solutions. (A) and (B) presented the RMSD values of sim-Na1 and sim-Na2, respectively.

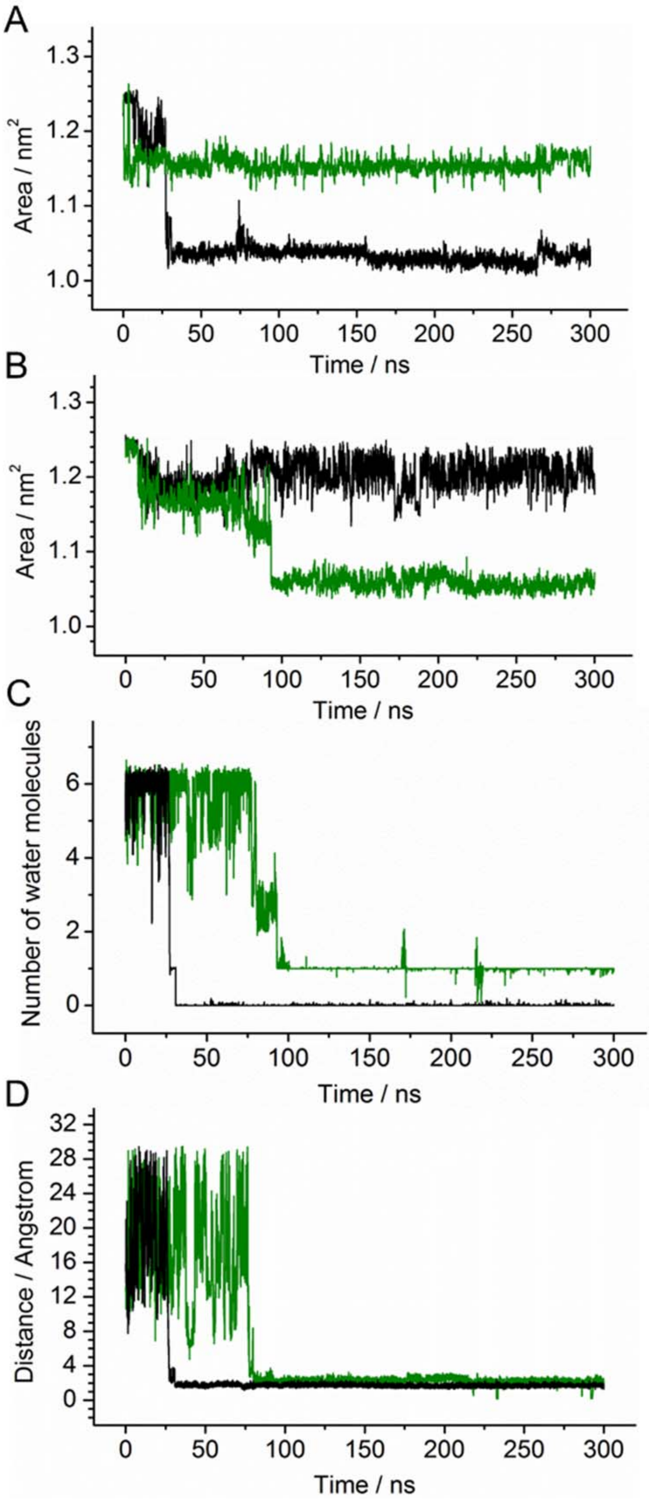

Figure S3

The variations of G-tetrad gate areas, coordinated waters and distances in the simulations of sim-Na1 and sim-Na2. (A) and (B) respectively showed the changes

of G-tetrad gate areas of sim-Na1 and sim-Na2, in which the top and bottom areas were colored black and green, respectively; (C) and (D) respectively presented the number of waters within 3.5 Å of binding Na^+ ions and the distances between the binding Na^+ ions and CCG in sim-Na1 (black) and sim-Na2 (green).
